# Graphene-Doped Polymethyl Methacrylate (PMMA) as a New Restorative Material in Implant-Prosthetics: In Vitro Analysis of Resistance to Mechanical Fatigue

**DOI:** 10.3390/jcm12041269

**Published:** 2023-02-06

**Authors:** Eduardo J. Selva-Otaolaurruchi, Lucía Fernández-Estevan, María Fernanda Solá-Ruiz, Fernando García-Sala-Bonmati, Inmaculada Selva-Ribera, Rubén Agustín-Panadero

**Affiliations:** 1 Department of Dental Medicine, Faculty of Medicine and Dentistry, University of Valencia, C/Gascó Oliag 1, 46010 Valencia, Spain; 2 Independent Researcher, 46010 Valencia, Spain

**Keywords:** graphene, PMMA, provisional, cad-cam, implants prostheses, cantilever

## Abstract

Background and Purpose: Provisional prostheses in restorations over several implants with immediate loading in completely edentulous patients increase the risk of frequent structural fractures. An analysis was performed of the resistance to fracture of prosthetic structures with cantilevers using graphene-doped polymethyl methacrylate (PMMA) resins and CAD-CAM technology. Methods: A master model was produced with four implants measuring 4 mm in diameter and spaced 3 mm apart, over which 44 specimens representing three-unit fixed partial prostheses with a cantilever measuring 11 mm were placed. These structures were cemented over titanium abutments using dual cure resin cement. Twenty-two of the 44 units were manufactured from machined PMMA discs, and 22 were manufactured from PMMA doped with graphene oxide nanoparticles (PMMA-G). All of the samples were tested in a chewing simulator with a load of 80 N until fracture or 240,000 load applications. Results: The mean number of load applications required for temporary restoration until the fracture was 155,455 in the PMMA-G group versus 51,136 in the PMMA group. Conclusions: Resistance to fracture under cyclic loading was three times greater in the PMMA-G group than in the PMMA group.

## 1. Introduction

Immediate loading in the case of full-arch prostheses in completely edentulous patients has been regarded as a safe and reliable technique, provided that primary implant stability is ensured, with passive fit, splinting and the elimination of micromovements capable of interfering with the implant osseointegration process [1,2]. Immediate loading allows the immediate restoration of chewing function, speech and aesthetics, with clear improvements to patient satisfaction and quality of life [3].

In these cases, the utilization over 3–6 months of a temporary prosthesis is considered to be necessary in order to complete the healing and osseointegration process [4,5]. Such provisional restorations are often made from polymethyl methacrylate (PMMA), due to its good properties. Classically, PMMA has been used as the material of choice, because of its simplicity of use and its low elastic modulus that avoids stress of the occlusal charge. PMMA has been employed using heat-curing techniques, though at present the material is used in the form of discs that can be machined using CAD-CAM technology, resulting in structures with better mechanical and biological properties [6,7,8].

Distal cantilevers are used in many of these provisional restorations in order to expand the occlusal surface. This may cause fractures of the restoration that pose a serious risk for implant stability (improve bone loss because during the occlusal charge; screw loosening), since they may result in an increase in micromovements that avoid/prevent osseointegration [9].

In order to avoid this problem, the addition of reinforcing materials to the main structure has been proposed, including metal beams, the incorporation of fibers and glass meshes, silica, carbon, polyamide, etc., [10,11,12,13]. One such reinforcing material is graphene, added to the composition of the PMMA discs in the form of small amounts of graphene particles. This material affords improved mechanical properties (fracture resistance) and has antibacterial activity with minimal cytotoxicity [14,15].

The main aim of the present study was to compare the performance and durability of samples of PMMA created with CAD-CAM technology and PMMA created with CAD-CAM technology doped with graphene oxide, regarding fatigue load across 240,000 cycles (12 months clinical life). The null hypothesis would be that the machined PMMA and the graphene-doped PMMA samples exhibited the same mechanical response.

## 2. Materials and Methods

Two titanium implants measuring 4 mm in diameter and 11.5 mm in length (BOST411 Zimmer Biomet, Palm Beach Gardens, FL, USA) were placed in a cylindrical nylon tube affixed with Exakto-Form^®^ epoxy resin (Bredent, Senden, Germany), following the specifications of standard UNE-EN ISO 14801:2017. The implants were spaced 3 mm apart, parallel to each other, and with an inclination of zero degrees.

**Figure 1:**
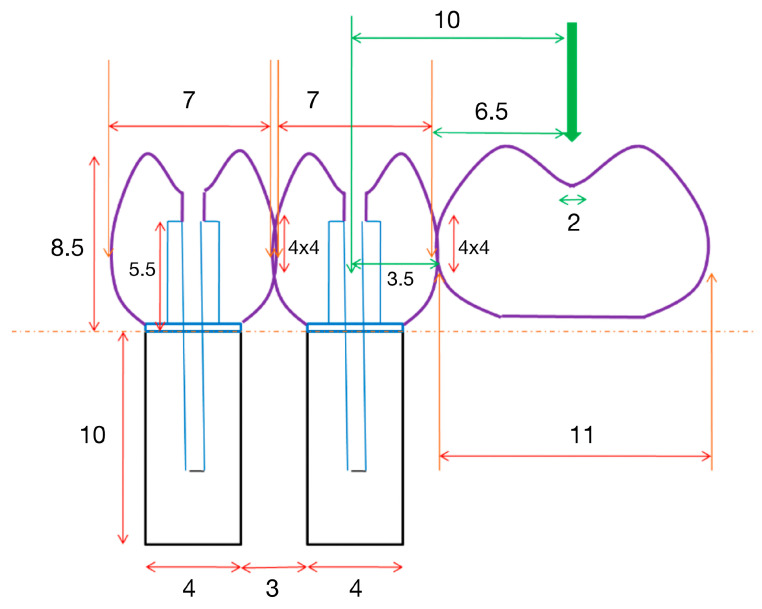
Specimen design. Measurements in millimeters.

Two premolar crowns measuring 7 mm in mesiodistal (M-D) width were designed. Likewise, a molar measuring 11 mm in M-D width was included in extension, joined distal to the crown over the implant by means of a 4 × 4 mm connector (Figure 1).

A total of 44 specimens were manufactured: 22 machined PMMA specimens (Huge PMMA Block, Huge Dental Material, Co., Ltd., Shanghai, China) (PMMA group) and 22 machined PMMA specimens doped with graphene (G-CAM, Graphenano Dental, Valencia, Spain) (PMMA-G group). A Datron C5 5-axes drill (Datron, Mühltal, Germany) was used.

Graphene nanofibers have diameters between 10 and 100 nanometers and lengths of 1000 nanometers. In transmission electron microscopy (TEM) analysis, they present a stacked cup structure, where the inside and outside of the fibers are exposed, with different heterogeneity. The chemical composition of graphene nanofibers was analyzed using X-ray photoelectron spectroscopy (XPS), and it was found that they are composed of 91% carbon, 2.5% silicon, and 6.5% oxygen. PMMA was doped with graphene in the range of 0.15–0.175% parts per million (Table 1). Graphene was dispersed into the resin monomer (liquid phase) by means of ultrasonic dispersion processes. The functional groups of graphene were opened to allow bonding by chemical bonds to the monomer (Table 2).

These milled structures with an access hole were bonded to titanium bases (Hexed FlexLink TiBase, Palm Beach Gardens, FL, USA) with dual-cured cement (RelyX Unicem 2 Automix, 3M ESPE, Seefeld, Germany), following a conventional protocol (titanium bases were sandblasted with 30 µm silica particles for 10 s). 

After curing the cement (auto- and photo-polymerized), the crowns were screwed onto the implants with a torque of 20 N cm, following the instructions of the manufacturer.

The tubes were stored in the saline solution until fatigue testing with a cyclic loading machine (Chewing Simulator CS-4.2, Mechatronik, Feldkirchen-Westerham, Germany). The simulator performed cycles of 15,000 applications of a load of 80 N to the center of the occlusal surface of the molar, 10 mm from the center of the most distal implant. Loading was applied by a steel ball affixed to the mobile axis of the machine and with a displacement of 2.5 mm. The simulator operated at a frequency of 2 Hz and with a vertical speed of 40 mm/s. No lateral loading was applied. The loading cycles were applied until fracture or the completion of 240,000 load applications, simulating 12 months of wear life, according to our immediate loading protocols.

An analysis was conducted of the variables time to fracture and length, with the calculation of the mean, standard deviation (SD), maximum, minimum, median and 25th and 75th percentiles.

The Kolmogorov–Smirnov test showed that the variable number of cycles did not exhibit a normal distribution; a nonparametric analytical approach was therefore adopted. The Mann–Whitney U-test was used for comparison of the maximum number of cycles between the two groups. The chi-square test was used to compare the fracture rates or the extent to which a certain threshold of load applications (120,000) was reached in the two study groups. The survival curves corresponding to the number of cycles applied until fracture were plotted using the Kaplan–Meier method, with the log-rank test for comparing the curves between the groups. The level of statistical significance was established as 5% (α = 0.05). Based on the Mann–Whitney U-test, with a confidence level of 95% and considering an effect size f = 1.8, the statistical power reached was 99.9% for the detection of statistically significant differences.

## 3. Results

Each provisional restoration underwent cyclic loading until fracture, recording the number of cycles at which fracture took place, or until the completion of 240,000 loads applications in the event of no fracture.

- In this regard, the specimens in the PMMA group recorded a median of 52,500 load applications (interquartile range [IQR]: 30,000–60,000) versus 120,000 (IQR: 120,000–240,000) in the PMMA-G group (Table 3).

**Table 3:** Maximum number of loading cycles according to group.

	GROUP		
	Total	PMMA	PMMA + Graphene
N	44	22	22
Mean	103,295.5	51,136.4	155,454.5
Standard deviation	72,928.3	22,516.2	68,433.3
Minimum	15,000.0	15,000.0	60,000.0
Maximum	240,000.0	120,000.0	240,000.0
Percentile 25	52,500.0	30,000.0	120,000.0
Median	60,000.0	52,500.0	120,000.0
Percentile 75	120,000.0	60,000.0	240,000.0

**Figure 2:**
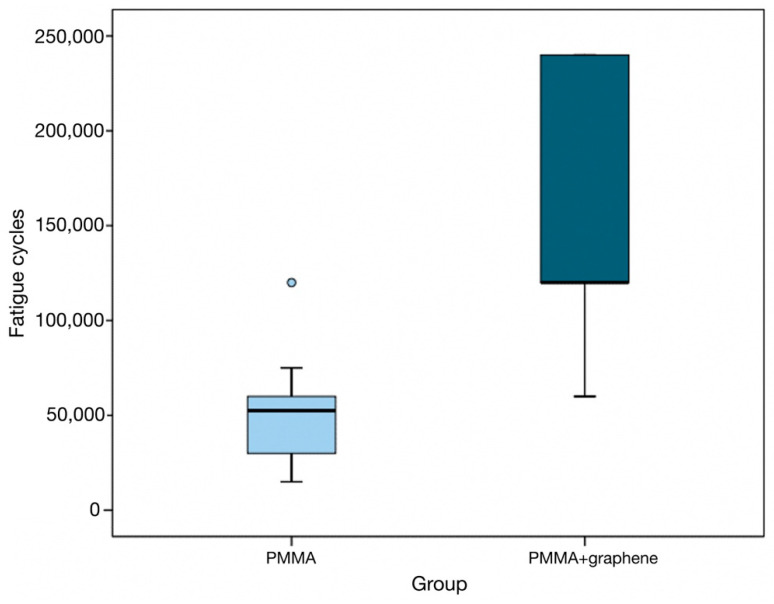
Maximum number of loading cycles according to group.

**Figure 3:**
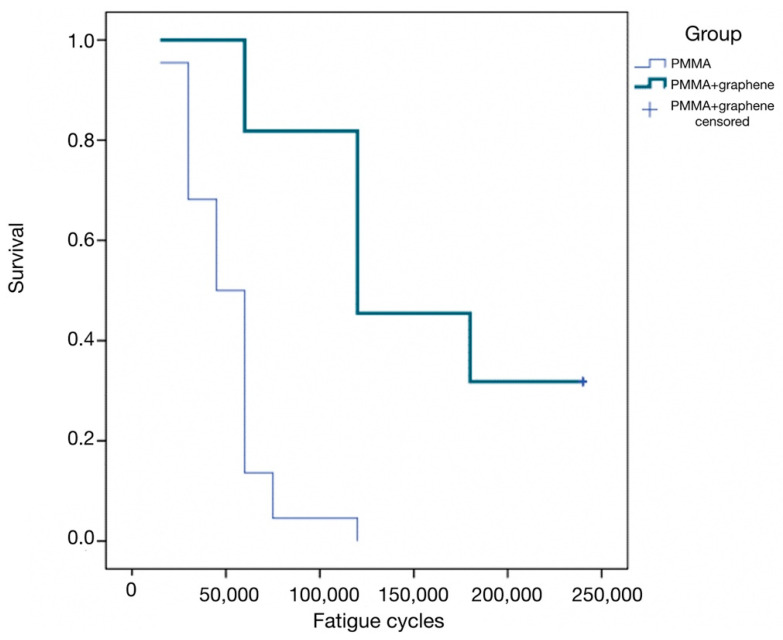
Kaplan–Meier survival curves corresponding to the number of loading cycles until fracture.

**Figure 4:**
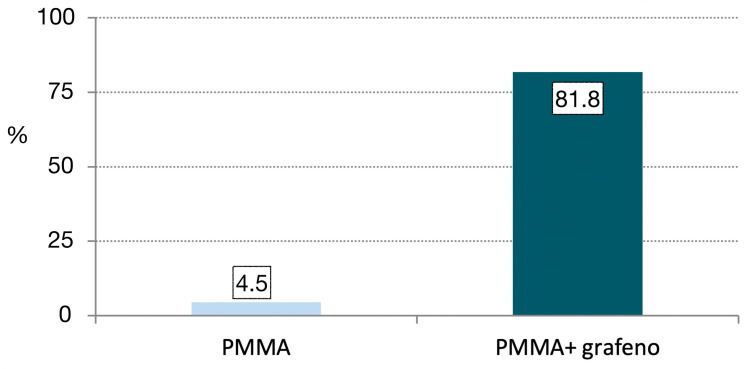
Incidence of restorations that resist 120,000 fatigue cycles.

**Figure 5:**
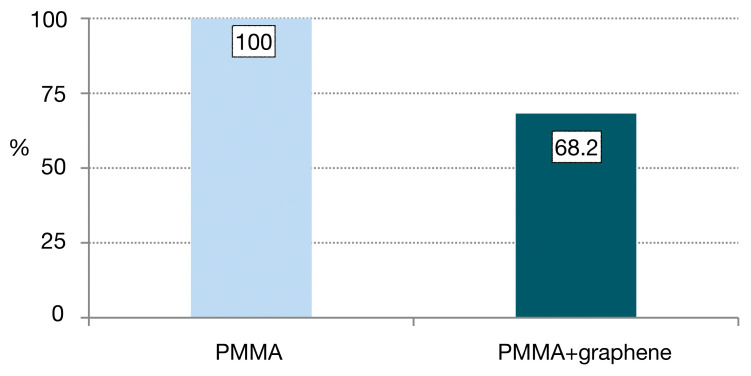
Incidence of fractures according to group.

- In descriptive terms, the difference between the two groups is significant, and the box plots show the number of cycles to be significantly greater in the graphene-doped specimen group (*p* < 0.001) (Figure 2).

- Likewise, the number of specimens that exceeded 120,000 load applications without fracture was significantly greater in the PMMA-G group than in the PMMA group (*p* < 0.001), and the corresponding fracture rate was significantly lower (*p* = 0.009).
- Lastly, the cumulative survival curves corresponding to the number of cycles up to fracture showed significant differences between the two groups (*p* < 0.001, log-rank test). Specifically, in the PMMA group, the median survival was 45,000 cycles (95% confidence interval [95%CI]: 33,508–56,491), versus 120,000 in the PMMA-G group (95%CI: 70,063–169,937) (Figure 3). This survival curve simulates a clinical situation, because the aim of the study was to analyze the resistance of this material in a fatigue load test. The great dispersion in the resistance values highlights the unpredictability of the behavior of the material, as it fractures at very different values. However, PMMA-G has, on average, better fracture resistance values.

- Most fractures occurred in both groups in the area of the junction with the titanium abutment, where the thickness of the PMMA was less than 2 mm (Figure 4 and Figure 5). The fatigue study was carried out either until the fracture of the material (which occurred in 100% of the PMMA samples) or up to 240,000 cycles, and 68.2% of the PMMA samples doped with graphene were fractured.

The results obtained show that from multiple perspectives, PMMA reinforced with graphene oxide proves effective in the manufacture of provisional restorations.

## 4. Discussion

The results obtained in this in vitro study indicate that provisional restorations manufactured with CAD-CAM technology and screwed over implants with cantilevers exhibit greater resistance to fractures when made from graphene-doped PMMA than when made from plain PMMA. This refutes our working hypothesis that the machined PMMA and graphene-doped PMMA samples exhibit the same mechanical response.

One of the materials used is graphene, which is added to the acrylic resin during the manufacturing process. Good homogenization of the graphene oxide within the PMMA matrix is crucial in this process in order to secure the best mechanical and biological properties, such as inhibition of the adherence of microorganisms. As a result of these characteristics, the use of graphene is recommended in dental prostheses and orthodontics [16,17,18].

The specimens used in the present study were intended to reproduce the clinical situation of a cantilever in a provisional restoration with immediate loading over implants. For this purpose, the specimens were designed to resemble a real-life prosthesis with anatomically shaped teeth [19].

**Figure 6:**
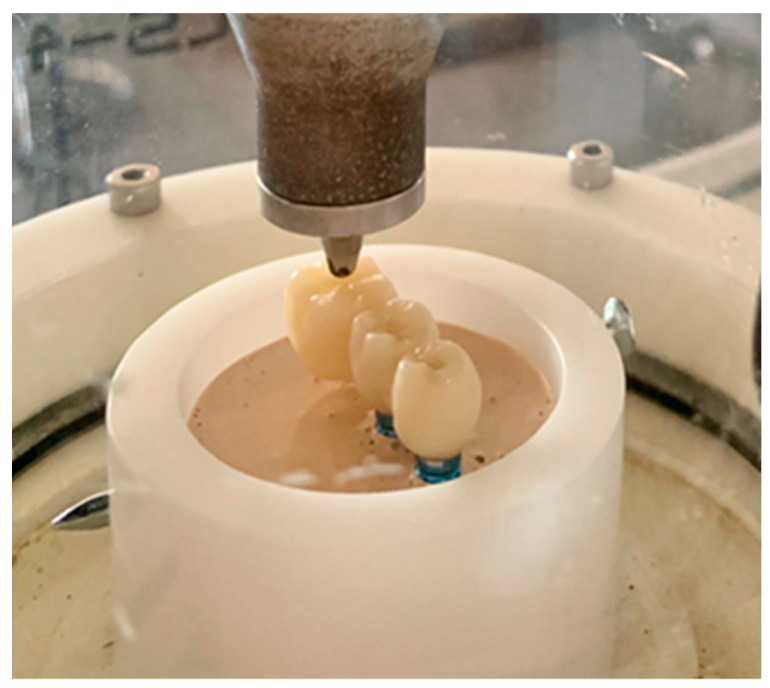
Study specimen mounted in the chewing simulator during fatigue testing.

Most authors do not perform tests on structures of this kind, but rather on bars of different shapes [20,21]. On the other hand, we applied occlusal loading to the center of the occlusal surface of the cantilever (a molar), 10 mm from the center of the most distal implant [22] (Figure 6).

**Figure 7:**
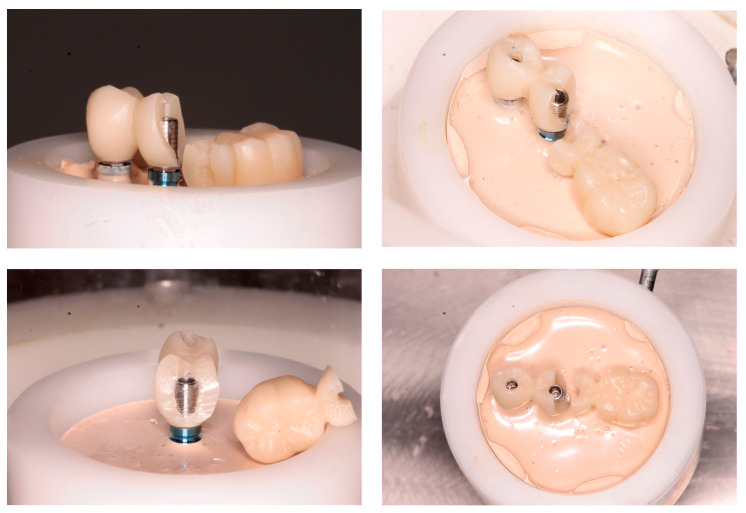
Fracture at the distal abutment level.

Although much has been written about the convenience or otherwise of cantilevers and their size [23,24], we decided to use a first molar, which reflects a frequent clinical scenario and affords a sufficient occlusal surface in the provisional prosthesis. The connector between the most distal abutment and the cantilever measured 4 × 4 mm, in order to simulate a minimum thickness similar to that recommended by Jemt [25]—though some authors consider that a greater thickness is needed in order to avoid fractures at this level [19] (Figure 7).

Aluminum implant replicas were used, since the mechanical resistance of this metal is far greater than that of acrylic resins, thus suggesting that they would have no impact upon the results obtained [26].

The implant replicas were placed in the nylon tubes of the chewing simulator using a splint for placement within the epoxy resin, in a stable and fully vertical position as recommended by standard ISO 14801:2017.

The applied load was 80 N, which is similar to the force used by Rosentritt [27] and represents a conventional occlusal force. This is an important but not a fundamental factor, since although some authors such as Suarez-Feito and Shen left the cantilevers free of occlusion, they still recorded an important incidence of fractures [24,28].

In general, there are two ways to produce immediate loading provisional prostheses. One approach is to use the full prosthesis of the patient, perforating where the implants and their provisional abutments have been positioned, and joining both structures with self-polymerizing acrylic resin. Then, appropriate trimming and polishing of the prosthesis can be carried out [29,30,31,32]. The alternative approach involves the obtainment of an impression at the time of surgery, with the manufacture of the PMMA within a few hours, joining it to provisional titanium abutments [33]. In the present study, we used this second approach for manufacturing the provisional restorations, because it allows the laboratory to produce the machined structures and perform bonding to the provisional abutments using cementing materials of greater quality. For such bonding, we used a technique similar to that described by Pitta, joining the provisional abutments to the PMMA and PMMA-G structure with dual cure resin following sandblasting with 30 µm silica particles at a pressure of 2 bar within the tubes of the acrylic structure [34]. This resulted in very stable bonding, which, in contrast to other studies such as that of Angelara et al., implied that no specimen was decemented [21].

These provisional restorations must remain in the mouth for several months, and in accordance with Soriano et al., we considered it important to know how they behave in response to fatigue after the repeated application of stress, thus leading us to perform cyclic fatigue testing. According to Steiner et al., 240,000 loading applications would be equivalent to one year of function in the mouth. As a result, the 6-month period during which the provisional restorations must remain in the maxilla would be represented by 120,000 load applications. In the case of the mandible, 60,000 applications would be representative of the three months of required presence in the mandible [35,36].

In clinical practice, there are several situations that require advanced surgical procedures with a high biological cost. In this in vitro study, we simulated an adverse clinical situation, where the limitations of bone availability require the use of prostheses with a distal cantilever [37].

The limitations of this study are the characteristics of an in vitro Test. This study, with low statistical power, is a preliminary study to analyze the behavior of these materials in an extreme situation such as distal cantilevers. PPMA-G proves to be a suitable material for use in the prosthesis; however, more clinical studies with a long follow-up period are necessary to analyze its biomechanical behavior.

## 5. Conclusions

The statistical data obtained in this in vitro study clearly reflect the benefits of PMMA doping with graphene oxide in the manufacture of immediate loading provisional restorations using CAD-CAM technology with a cantilever molar. Despite the solidness of the results, since this is an in vitro study, caution is required in extrapolating the findings to a real-life clinical setting. Furthermore, the data obtained should be complemented by thermocycling studies.

## Figures and Tables

**Table 1:** Percentage surface composition of graphene nanofibers. Deconvolution of high-resolution XPS photopeaks.

Phototopic	Binding Energy (eV)	Binding Type	Percentage (% at)
C1s	284.5	C-C, C=C	84
	285.9	C-O	10
	287.2	C=O	4
	290	COOR	2
O1s	532	C=O	19
	533.8	C-O	52
	535.3	OH	29
Si2p	104	SiO_2_	46
	105.5	Si-O	54

**Table 2:** Main characteristics of graphene nanofibers.

Characteristics	Properties	Graphene Nanofibers (GNF)
Characteristics Textural	Surface area (m^2^/g)	70–250
	Micropore area (m^2^/g) ^a^	2–50 (2–20)
	Total pore volume (cm^3^/g)	0.3–1.6
Degree of Graffiti	DRX: npg ^b^	10–25 (npg of graphite ≈ 95)
	Raman: ID/IG ^c^	0.95–1.05 (ID/of graphite ≈ 0.6)
Characteristics Physical/Chemical	Fiber diameter (nm) ^d^	5–160
	Fiber length (nm) ^d^	>20
	Catalyst content (%) (CNF gross, unpurified)	12–20
	Elemental analysis of the product (free of catalyst residues) (% mole)	C (75–93) O (2.5–22) H (4.5–5.5)
Characteristics Thermal	Oxidation temperature (°C) ^e^	350–680 (520–640)
	Decomposition products/Thermal oxidation	CO, CO_2_ mainly

^a^ In parentheses: % of micropore area with respect to the total surface area. ^b^ Number of graphene planes in the crystal (npg = Lc/d002); D002 is the interlaminar spacing; Lc is the average size of the crystals in the direction perpendicular to the basal graphene planes. ^c^ ID/IG: ratio between the intensities of the D and G bands in the Raman spectrum. ^d^ Determined by counting at least 200 GNFs in electron microscopy micrographs transmission. ^e^ In parentheses: range of temperatures corresponding to the maximum oxidation.

## Data Availability

Not applicable.
